# Evaluation and Selection of *Rubus* spp.× *Rubus chingii* Hybrids with Excellent Overall Fruit Quality and High Drought Tolerance

**DOI:** 10.3390/plants15060899

**Published:** 2026-03-13

**Authors:** Yue Li, Yiru Zhang, Yaqiong Wu, Zhengjin Huang, Lianfei Lyu, Weilin Li, Chunhong Zhang

**Affiliations:** 1 Jiangsu Key Laboratory for Conservation and Utilization of Plant Resources, Institute of Botany, Jiangsu Province and Chinese Academy of Sciences (Nanjing Botanical Garden Mem. Sun Yat-Sen), Nanjing 210014, China; lly020401@163.com (Y.L.); zhangyiru79@163.com (Y.Z.); hzj90@cnbg.net (Z.H.); njbglq@163.com (L.L.); 2 State Key Laboratory for Development and Utilization of Forest Food Resources, Nanjing Forestry University, 159 Longpan Road, Nanjing 210037, China; wlli@njfu.edu.cn

**Keywords:** blackberry, interspecific hybridization, drought tolerance, inherited tendency, principal component analysis

## Abstract

Blackberry cultivars typically exhibit high fruit antioxidant levels but poor drought tolerance compared with their wild *Rubus* relatives. Few studies have employed wild *Rubus* species in hybridization programs aimed at improving drought tolerance and fruit quality in cultivated blackberries. In this study, we comprehensively assessed growth traits, fruit characteristics, and drought tolerance in 108 F_1_ progenies derived from a cross between the cultivated blackberry ‘Prime-Ark^®^ Freedom’ and the wild species *Rubus chingii*. Correlation analysis of fruit morphological traits indicated significant positive associations among single fruit weight, fruit thickness, and fruit diameter, reflecting coordinated fruit development. Among the nutritional quality traits evaluated, both anthocyanin and total phenolic contents exhibited transgressive segregation. Specifically, 47.78% of the progeny demonstrated higher anthocyanin content, and 45.56% exhibited greater total phenolic content than the higher-performing parent. The corresponding genetic transmission ability (Ta) reached 139.23% and 101.24% for these traits, respectively, indicating pronounced additive genetic effects and high heritability. After a 7-day drought treatment, the hybrid progenies exhibited significant heterosis in catalase (CAT) activity, with 24.07% exceeding the higher-parent value. In contrast, proline content exhibited high broad-sense heritability (H^2^ = 0.990) and considerable genetic variation. Under drought stress, all chlorophyll components were strongly positively correlated. Using principal component analysis (PCA), we established comprehensive evaluation models for fruit quality and drought tolerance. Based on these models, seven accessions—H3, H4, H8, H10, H11, H14, and H25—were identified as superior in both drought tolerance and fruit quality. This study provides an integrated evaluation framework for selecting drought-tolerant and high-quality genotypes from interspecific hybrid progenies in blackberry, offering a theoretical basis for utilizing wild *Rubus* resources in breeding improved cultivars.

## 1. Introduction

Blackberry (*Rubus* spp.) is a perennial shrub and an economically significant small fruit crop [1]. Although blackberry fruits are valued for their bioactive compounds, such as anthocyanins and polyphenols with antioxidant properties [2,3], their commercial appeal—particularly for the fresh market—is often limited by high organic acid levels, which result in excessive sourness, and a restricted aromatic profile in many cultivars [4,5].

This sensory limitation, combined with growing consumer demand for superior flavor, underscores the urgent need for breeding strategies that simultaneously enhance fruit quality and stress tolerance. Among environmental stresses, drought is a major constraint; most cultivated blackberries require high soil moisture (>85%), and growth is severely inhibited when soil moisture drops to 40–50%. Previous research on drought tolerance has focused on a limited number of cultivars, including ‘Hull Thornless’, ‘Ningzhi 1’, and ‘Kiowa’ [6,7,8,9], and has primarily characterized physiological responses such as osmotic adjustment. Consequently, the genetic architecture underlying drought tolerance in blackberry remains poorly understood.

This knowledge gap is exacerbated by the narrow genetic base of homoploid cultivars, a result of their recent and closely related origins, which significantly hinders breeding progress [10,11]. Interspecific hybridization with wide relatives offers a strategic approach to overcoming this genetic bottleneck, enabling the introgression of valuable resistance and flavor genes from wild germplasm into elite cultivars [12]. For instance, wild Mexican blackberry (*R. trivialis*) has been successfully used in breeding commercial cultivars such as ‘Ouachita’ and ‘Navaho’, which combine strong adaptability—including drought and heat tolerance—with desirable horticultural traits [13].

Among wild *Rubus* species, *Rubus chingii* stands out as a particularly valuable donor. Its mature fruits are valued not only for their delicate texture and nutritional richness but also for their considerable medicinal properties [14]. Compared to cultivated blackberries, *Rubus chingii* fruits exhibit higher titratable acidity and a more layered, complex flavor profile [15]. The species also exhibits strong drought tolerance, thriving in soils with 40–60% water content [16]. Previous studies have highlighted interspecific hybridization with such wild germplasm as a promising strategy for berry improvement [17]. Thus, introgressing the drought tolerance and flavor traits of *Rubus chingii* into cultivated blackberry holds significant practical value for climate-adaptive breeding [18].

Despite the recognized potential of wild *Rubus* germplasm, systematic evaluations of interspecific F_1_ hybrid populations—particularly those integrating comprehensive assessments of both agronomic performance and drought tolerance—remain scarce. In this study, we utilized an F_1_ population derived from a cross between the cultivated blackberry ‘Prime-Ark^®^ Freedom’ and the wild species *Rubus chingii* to: (1) analyze genetic variation and heritability of key agronomic and physiological traits; (2) examine correlations among fruit quality and drought tolerance traits to understand potential trade-offs; and (3) identify superior hybrids with balanced performance using principal component analysis.

## 2. Results

### 2.1. Genetic Analysis of Growth Traits in Hybrid Progeny of Rubus *spp.* × Rubus chingii 

#### 2.1.1. Leaf Morphological Traits

**Figure 1:**
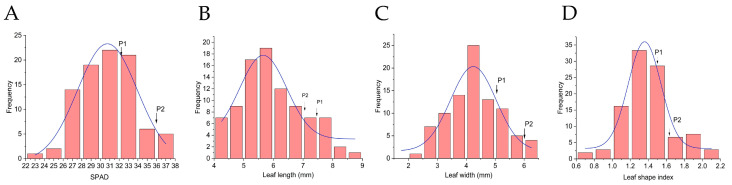
Frequency distribution of leaf traits in the F_1_ population. (**A**) SPAD value; (**B**) Leaf length; (**C**) Leaf width; (**D**) Leaf shape index.

Continuous variation was evident across all measured leaf traits in the hybrid progeny. The leaf SPAD value, with its relatively low broad-sense heritability (H^2^ = 0.512), pointed to substantial environmental influence on phenotypic expression (Figure 1A). For leaf length, the progeny mean (5.88 cm) fell significantly below the mid-parent value (7.27 cm), yielding a genetic transmission ability (Ta) of 80.88%; remarkably, however, its heritability reached 0.993 (Figure 1B). A similar pattern emerged for leaf width: the mean (4.29 cm) was lower than the MP (5.60 cm) with Ta = 76.61%, yet heritability remained comparably high (H^2^ = 0.984) (Figure 1C). Leaf shape index presented a contrasting picture: the population mean (1.41) not only exceeded the MP (1.34) but also skewed toward the maternal parent (Figure 1D). With both high broad-sense heritability (H^2^ = 0.991) and strong genetic transmission ability (Ta = 105.22%), this trait exemplified predominantly genetic control (Table 1).

#### 2.1.2. Floral Organ Traits

**Figure 2:**
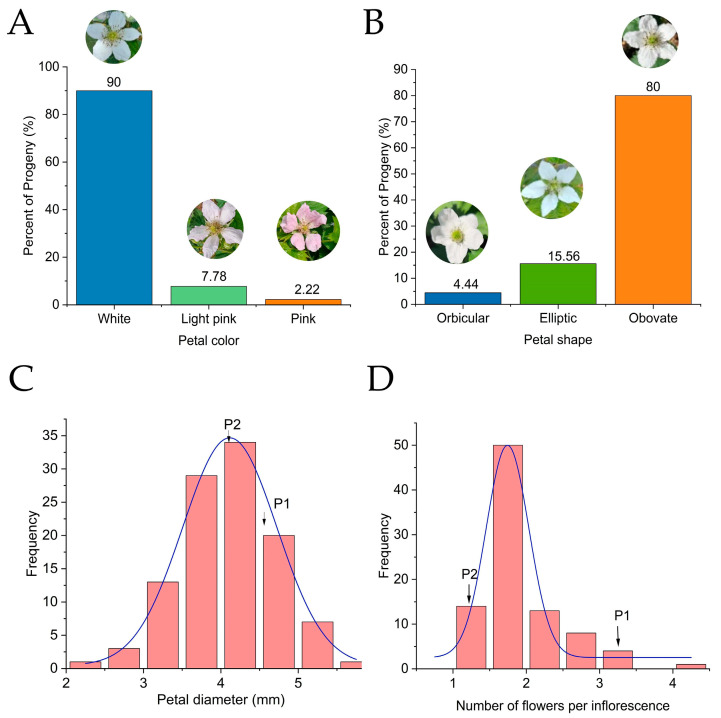
Frequency distribution of floral traits in the F_1_ population. (**A**) Petal color; (**B**) Petal shape; (**C**) Petal diameter; (**D**) Number of flowers per inflorescence.

With vegetative traits assessed, attention turned to floral characteristics within the hybrid population. Flower color segregated into three distinct phenotypes—white (90.0%), light pink (7.78%), and pink (2.22%)—while petal shape similarly partitioned into orbicular (4.44%), elliptic (15.56%), and obovate (80.0%) types (Figure 2A,B). Quantitatively, mean petal diameter (40.8 mm) closely tracked the mid-parent value (43.0 mm), its high broad-sense heritability (H^2^ = 0.987) pointing to strong genetic determination (Figure 2C). Flowers per inflorescence told a different story: the progeny mean (1.61) fell substantially below the MP (2.25) (Figure 2D). With low heritability (H^2^ = 0.488), high coefficient of variation (37.68%), and a mere 0.93% of progeny exceeding the higher-parent value, this trait appeared predominantly shaped by environmental conditions rather than genetic factors (Table 1).

### 2.2. Genetic Analysis of Fruit Traits in Hybrid Progeny of Rubus *spp.* × Rubus chingii

#### 2.2.1. Appearance Quality Traits

**Figure 3:**
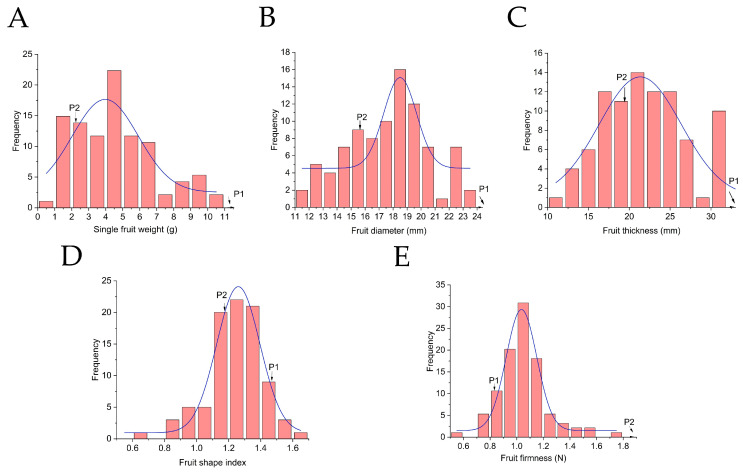
Frequency distribution of fruit appearance traits in the F_1_ population. (**A**) Single fruit weight; (**B**) Fruit diameter; (**C**) Fruit thickness; (**D**) Fruit shape index; (**E**) Fruit firmness.

Fruit appearance traits showed a consistent trend: progeny means for single fruit weight (SFW), fruit diameter (FD), and fruit thickness (FT) all fell significantly below their respective mid-parent values. Among these, SFW registered the lowest genetic transmission ability (Ta = 53.97%). For both SFW and FT, no progeny surpassed the higher-parent value, with 58.33–71.43% occupying intermediate positions. Broad-sense heritability estimates, ranging from 0.694 to 0.825, pointed to predominantly genetic control (Figure 3A–C).

Fruit shape index departed from this pattern, displaying positive heterosis in the F_1_ generation: the population mean (1.37) exceeded the MP (1.32) with Ta = 103.79%. Despite moderate heritability and appreciable phenotypic variation, intermediate types dominated the distribution, though both high- and low-parent types were also represented (Figure 3D).

Fruit firmness presented yet another pattern. With an MP of 1.64 N and a progeny mean of only 1.04 N, the vast majority (90.11%) fell between the two parents, and none exceeded the higher-parent value. Its low broad-sense heritability (H^2^ = 0.325) pointed to substantial environmental influence (Figure 3E; Table 2).

#### 2.2.2. Nutritional Quality Traits

**Figure 4:**
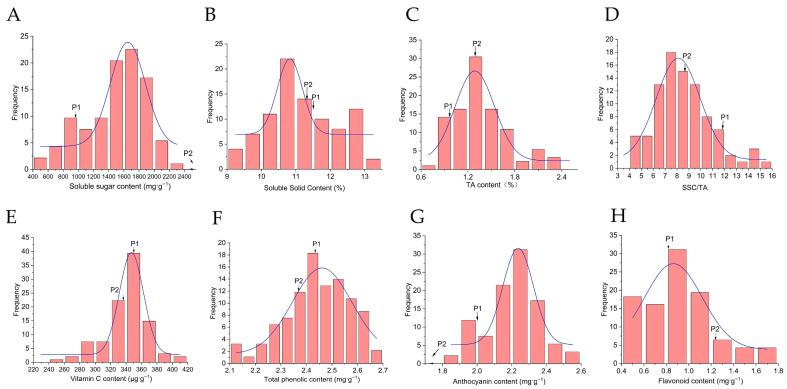
Frequency distribution of fruit quality traits in the F_1_ population. (**A**) Soluble sugar content; (**B**) Soluble solids content; (**C**) TA content; (**D**) SSC/TA; (**E**) Vitamin C content; (**F**) Total phenolic content; (**G**) Anthocyanin content; (**H**) Flavonoid content.

Continuous variation, a hallmark of polygenic quantitative inheritance, characterized all measured fruit quality traits. Between the parental lines, soluble sugar content differed substantially. The progeny mean (1499.94 μg·g^−1^) fell markedly below the mid-parent value (MP, 2583.15 μg·g^−1^), yielding a genetic transmission ability (Ta) of just 58.07%. Notably, no progeny surpassed the higher-parent value; instead, 85.56% occupied intermediate phenotypes, with the frequency distribution peaking squarely within the parental range (Figure 4A).

Soluble solids content presented a more complex picture. While the population mean (11.19%) dipped only slightly below the MP (11.42%), the trait exhibited wide segregation: 34.44% of progeny exceeded the higher parent, and 54.44% fell below the lower parent (Figure 4B). Titratable acidity followed yet another pattern. Progeny values (1.41%) significantly outstripped the MP (1.14%), accompanied by a high Ta (123.68%) and 57.78% of progeny surpassing the higher-parent value (Figure 4C).

The solid–acid ratio (SSC/TA), a key determinant of flavor, registered exceptionally high broad-sense heritability (H^2^ = 0.999). Yet its population mean (8.51) fell substantially below the MP (10.25), with 58.89% of progeny distributed below the lower parent and a mere 8.89% surpassing the higher parent (Figure 4D).

Vitamin C content told a different story. Comparable to the MP (340.11 vs. 343.94 μg·g^−1^), it exhibited both high Ta (98.98%) and extremely high broad-sense heritability (H^2^ = 0.998)—clear evidence of strong genetic stability. Moreover, 27.78% of progeny exceeded the higher-parent value, pointing to additive gene effects as the predominant governing mechanism (Figure 4E).

Both anthocyanin and total phenolic contents displayed transgressive segregation. Their mean values surpassed the respective MPs, with 45.56% (total phenolics) and 47.78% (anthocyanins) of progeny exceeding the higher-parent value. The very high broad-sense heritability recorded for both traits underscored strong genetic control and considerable potential for selecting superior genotypes (Figure 4F–G).

Flavonoid content presented yet another pattern. The progeny mean (0.93 mg·g^−1^) fell below the MP (1.04 mg·g^−1^) while maintaining high broad-sense heritability (Figure 4H). Notably, the phenotypic distribution skewed toward the maternal parent (P1), implicating maternal genetic background as a dominant contributor to flavonoid accumulation (Table 3).

### 2.3. Fruit Trait Correlation and Comprehensive Evaluation in Rubus *spp.* × Rubus chingii 

#### 2.3.1. Correlation Analysis of Fruit Traits

**Figure 5:**
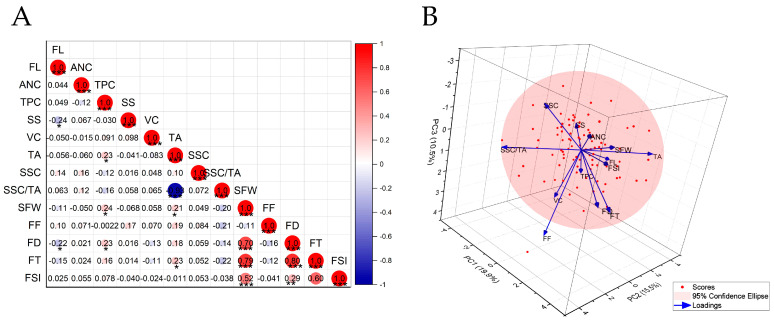
Correlation analysis and three-dimensional PCA biplot of fruit traits. (**A**) Correlation heatmap. Red indicates a positive correlation, blue indicates a negative correlation; color intensity and circle size correspond to the magnitude of the correlation coefficient. *, **, and *** denote significance at *p* < 0.05, 0.01, and 0.001, respectively. (**B**) PCA biplot. The spheres represent individual F_1_ plants; the arrows represent trait loadings, with the direction indicating the correlation to each principal component. The percentages on the axes indicate the variance explained by PC1, PC2, and PC3. FL, flavonoid content; ANC, anthocyanin content; TPC, total phenolic content; SS, soluble sugar content; VC, vitamin C content; TA, titratable acid content; SSC/TA, solid–acid ratio; SSC, soluble solids content; SFW, single-fruit weight; FF, fruit firmness; FSI, fruit shape index; FD, fruit diameter; FT, fruit thickness.

Pearson correlation analysis of the 13 measured fruit traits revealed distinct patterns among appearance and nutritional quality indices (Figure 5A). For morphological traits, single fruit weight (SFW) emerged as a central hub, exhibiting significant positive correlations with fruit thickness (FT), fruit diameter (FD), and fruit shape index (FSI). This interrelationship among size- and shape-related components underscores their coordinated development.

Turning to flavor-related traits, a striking negative correlation emerged between titratable acidity (TA) and the solid–acid ratio (SSC/TA). TA also showed positive associations with both SFW and FT, pointing to a tendency for larger fruits to carry higher acidity. SSC/TA, by contrast, correlated negatively with FT—a relationship suggesting that more elongated fruits may compromise sugar–acid balance.

Secondary metabolites revealed yet another layer of complexity. Total phenolic content (TPC) tracked positively with SFW, TA, and FD, indicating that larger fruits accumulate greater quantities of phenolics. Flavonoid (FL) content, however, painted a contrasting picture: significant negative correlations with both soluble sugar (SS) content and FD pointed to a potential trade-off in resource allocation between flavonoid and sugar biosynthesis.

#### 2.3.2. Principal Component Analysis of Fruit Traits

Principal component analysis (PCA) applied to the 13 fruit traits identified four primary dimensions underlying fruit quality (Figure 5B). Together, the first four principal components (PCs) explained 55.96% of the total variance, capturing the essential structure of the original dataset. The first component (PC1) integrated fruit size with fundamental acidity. Loading heavily on fruit thickness, fruit diameter, fruit shape index, and single fruit weight—while also correlating positively with titratable acidity and negatively with the solid–acid ratio—PC1 effectively encapsulated a size–acidity composite factor. Flavor balance emerged as the dominant theme of PC2. This component showed its strongest association with the solid–acid ratio, accompanied by negative loadings on titratable acidity and flavonoid content, thereby capturing the core sugar–acid equilibrium. PC3 linked textural attributes with nutritional quality. Characterized by strong positive loadings on fruit firmness and vitamin C content, this component was accordingly designated a texture–nutrient factor. The fourth component (PC4) highlighted the relationship between phenolic accumulation and fruit size. With high positive loadings on total phenolic content and single fruit weight, coupled with a negative loading on flavonoid content, PC4 pointed to a distinct phenolic accumulation dimension (Table 4).

#### 2.3.3. Evaluation and Grading of Individual Plants Based on Fruit Traits

The weight of each principal component was calculated according to the formula

$$w_{k}=\frac{\lambda_{k}}{\sum_{t=1}^{4}\lambda_{t}},yielding:w_{1}\approx 0.3563;w_{2}\;\approx 0.2768;w_{3}\approx 0.1868,w_{4}\;\approx 0.1801.$$

A comprehensive fruit quality evaluation model was constructed as follows:

F= 0.3563 × F1+0.2768 × F2+ 0.1868 × F3+ 0.1801 × F4

where *F*1 to *F*4 are the scores of the first four principal components. The model was applied to evaluate the fruit quality of the 90 hybrids. Comprehensive scores ranged from –1.923 to 1.705. Based on the distribution of these scores, individuals were classified into four fruit quality grades, superior, good, medium, and poor, accounting for 20%, 30%, 30%, and 20% of the population, respectively. Following this evaluation, the plants were grouped according to their assigned quality grade (Table 5).

### 2.4. Genetic Analysis of Drought Tolerance in Rubus *spp.* × Rubus chingii Hybrids 

Drought tolerance was comprehensively evaluated by subjecting all 108 F_1_ individuals to drought stress and analyzing inheritance patterns across key physiological traits.

**Figure 6:**
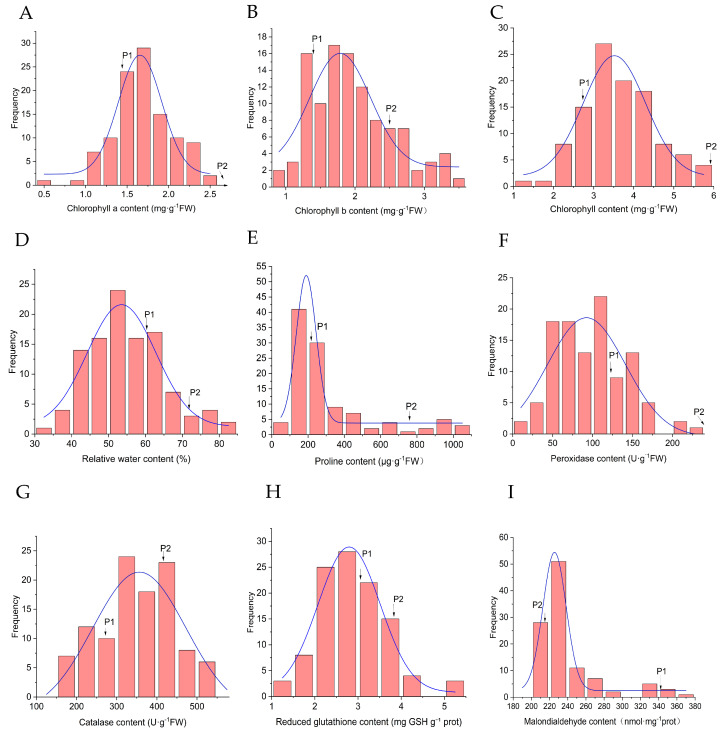
Frequency distribution of drought tolerance traits in the F_1_ population. (**A**) Chlorophyll a content; (**B**) Chlorophyll b content; (**C**) Chlorophyll content; (**D**) Relative water content; (**E**) Proline content; (**F**) Peroxidase activity; (**G**) Catalase activity; (**H**) Content of reduced glutathione; (**I**) Malondialdehyde content.

Among photosynthetic parameters, chlorophyll components revealed markedly distinct inheritance behaviors. Mean chlorophyll b (Chl b) content (1.98 mg·g^−1^ FW) slightly exceeded the mid-parent value (1.93 mg·g^−1^ FW), yielding a genetic transmission ability (Ta) of 102.77%—evidence of partial heterosis, with 18.52% of progeny surpassing the higher-parent value. Total chlorophyll and chlorophyll a (Chl a) told a different story: both fell below their respective MPs (Ta = 84.12% and 68.46%), yet maintained high broad-sense heritability (H^2^ = 0.902 and 0.749). The absence of transgressive segregation in these traits pointed to predominantly additive genetic control (Figure 6A–C).

Leaf relative water content (RWC) presented yet another pattern. The F_1_ mean (54.91%) dropped substantially below the MP (65.93%), registering a Ta of 83.30% and heritability of 0.858. Though 6.48% of individuals exceeded the higher parent, the majority (68.52%) fell below the lower parent—a clear shift toward the lower parent. Despite this, RWC retained relatively high genetic stability (Figure 6D).

Osmotic adjustment, assessed through proline (Pro) content, revealed striking contrasts. One parent exhibited notably high proline (758.57 μg·g^−1^ FW), yet the F_1_ mean (322.55 μg·g^−1^ FW) fell substantially below the MP (488.17 μg·g^−1^ FW) with a modest Ta of 66.07% (Figure 6E). Paradoxically, this trait displayed remarkably high broad-sense heritability (H^2^ = 0.990) and considerable genetic variation (CV = 88.04%).

The antioxidant system yielded equally diverse inheritance patterns. Peroxidase (POD) activity exemplified low-parent inheritance: the F_1_ mean (97.34 U·g^−1^ FW) significantly underperformed both MP and lower-parent values, with low Ta (34.94%) and no progeny exceeding the higher parent (Figure 6F). Catalase (CAT) activity, by contrast, slightly exceeded the MP (350.70 vs. 344.29 U·g^−1^ FW), accompanied by a Ta of 101.86% and high heritability (H^2^ = 0.976). Notably, 24.07% of progeny surpassed the higher parent—clear evidence of heterosis (Figure 6G). Reduced glutathione (GSH) content leaned toward the lower parent (mean 2.90 vs. MP 3.44 mg GSH·g^−1^ prot), yet 14.81% of individuals exceeded the higher parent, with the trait maintaining high heritability (H^2^ = 0.922) and a substantial Ta (84.36%) (Figure 6H).

Malondialdehyde (MDA) content, a marker of membrane lipid peroxidation, registered slightly below the MP (242.83 vs. 278.43 nmol·mg^−1^ prot). High Ta (87.22%) and heritability (H^2^ = 0.907) suggested primarily additive genetic control (Figure 6I; Table 6).

### 2.5. Correlation Analysis and Comprehensive Evaluation of Drought-tolerance Traits in Rubus *spp.* × Rubus chingii Hybrids

#### 2.5.1. Correlation Analysis

**Figure 7:**
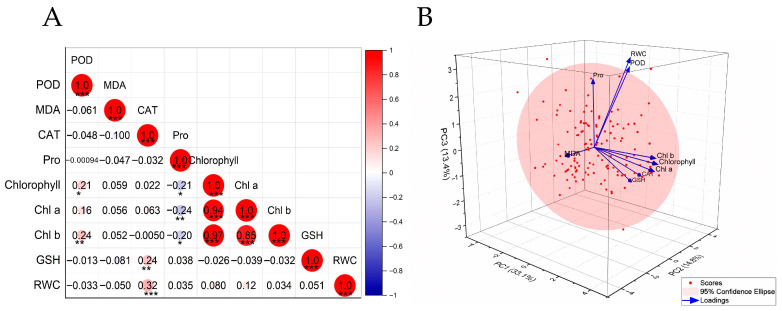
Correlation analysis and PCA biplot of drought tolerance traits. (**A**) Correlation heatmap. Red indicates a positive correlation, blue indicates a negative correlation; color depth and circle size correspond to the strength of the correlation coefficient. *, **, and *** denote significance at *p* < 0.05, 0.01, and 0.001, respectively. (**B**) PCA biplot. The spheres represent individual F_1_ plants; the arrows represent trait loadings, with the direction indicating their correlation to each principal component. The percentage of variance explained by PC1, PC2, and PC3 is indicated on the respective axes. POD, peroxidase activity; MDA, malondialdehyde content; CAT, catalase activity; Pro, proline content; Chlorophyll, total chlorophyll content; Chl a, chlorophyll a content; Chl b, chlorophyll b content; GSH, reduced glutathione content; RWC, relative water content.

Pearson correlation analysis of physiological indices under drought stress (Figure 7A) uncovered several significant trait associations that illuminate the complex stress response network. The photosynthetic components—total chlorophyll, chlorophyll a, and chlorophyll b—formed a tightly coordinated cluster, their strong positive intercorrelations pointing to synchronized regulation in the face of water deficit.

Within the antioxidant and osmoregulatory systems, a different pattern emerged. Catalase (CAT) activity showed positive associations with both reduced glutathione (GSH) content and leaf relative water content (RWC), suggesting that synergistic antioxidant activity helps sustain leaf water status under stress. Proline (Pro) content, however, painted a contrasting picture: its negative correlation with chlorophyll a hinted at a potential trade-off—resources diverted toward osmotic adjustment may come at the expense of photosynthetic function during drought.

#### 2.5.2. Principal Component Analysis

Principal component analysis (PCA) applied to physiological traits under drought stress extracted four principal components (PCs) that together explained 72.16% of the total variance, effectively capturing the essential dimensions of drought-response variation among the hybrid progeny (Figure 7B).

The component loadings, detailed in Table 7, revealed distinct functional groupings. PC1 captured overall photosynthetic capacity, with high positive loadings on total chlorophyll, chlorophyll a, and chlorophyll b. PC2 loaded heavily on catalase (CAT) activity, reduced glutathione (GSH) content, and leaf relative water content (RWC)—a combination reflecting integrated antioxidant and water-retention capacity. PC3 linked osmotic adjustment with reactive oxygen species (ROS) scavenging, showing strong associations with RWC, proline (Pro) content, and peroxidase (POD) activity, all crucial for maintaining cellular water balance. Together, these four components delineated the primary physiological axes underlying drought response in the hybrid population.

#### 2.5.3. Drought Tolerance Evaluation Score and Individual Plant Grading

On the basis of the variance contributions in Table 7, the weight $w_{k}$ for each principal component was calculated as follows: $w_{1}\;$≈ 0.5403, $w_{2}\;$≈ 0.2406, and, similarly, $w_{3}\;$≈ 0.2191. A comprehensive drought tolerance evaluation model was constructed as follows:

$$F_{n}\;=\;0.5403Y_{1}\;+\;0.2406Y_{2}\;+\;0.2191Y_{3},$$

where $Y_{1}$ to $Y_{3}$ represent the scores of the first four principal components. Implementation of this model on the 108 hybrid progenies yielded scores ranging from –2.002 to 2.571. Based on the score distribution, plants were classified into four drought tolerance categories: highly tolerant (22 plants), moderately tolerant (32 plants), moderately sensitive (32 plants), and highly sensitive (22 plants).

### 2.6. Selection of Elite Progenies for Combined High Fruit Quality and Drought 

**Figure 8:**
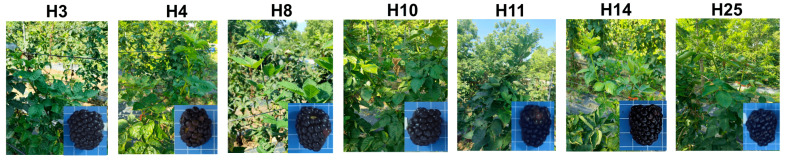
Phenotypic performance of seven elite single plants combining high fruit quality and drought tolerance.

From the integrated evaluation of fruit quality (Section 2.3.3) and drought tolerance (Section 2.5.3), seven elite individuals emerged—H3, H4, H8, H10, H11, H14, and H25. Selection criteria were twofold: only those genotypes ranking in the top 20% (Superior grade) of the fruit quality model and simultaneously classified as “highly tolerant” (top 20%) within the drought tolerance model were considered elite (Figure 8).

These accessions uniquely combine high drought tolerance with superior fruit quality, positioning them as promising candidates for breeding programs focused on concurrently enhancing stress resilience and horticultural traits. Their exceptional performance across key traits is presented in Table 8.

## 3. Discussion

The parental lines selected for this study—cultivated blackberry ‘Prime-Ark^®^ Freedom’ and its wild relative *Rubus chingii*—exhibited pronounced divergence in both agronomic performance and drought tolerance, making them ideal candidates for investigating trait inheritance. Their F_1_ progeny displayed wide phenotypic segregation, with extreme phenotypes spanning multiple traits, thereby providing a rich resource for elite genotype selection and cultivar development. Systematic evaluation of growth, fruit quality, and drought-response traits has yielded a phenotypic and genetic foundation to guide targeted blackberry breeding.

Vegetative traits in the F_1_ population—leaf width, length, and shape index—fell predominantly under genetic control. Notably, most plants exhibited leaf morphology resembling the maternal parent rather than the palmate compound leaves characteristic of the paternal line, pointing to additive genetic effects with possible modulation by maternal cytoplasmic inheritance [19]. Leaf SPAD value and inflorescence number per branch, by contrast, registered low heritability, implicating environmental factors—light, water, and nutrient availability—as primary drivers of phenotypic variation in these traits (Table 1) [20]. Multi-environment trials, potentially coupled with genomic selection, thus emerge as logical next steps for improving selection efficiency [21].

Fruit appearance and commercial value hinge critically on fruit thickness, fruit diameter, and single fruit weight [22]. Here, all three traits displayed quantitative inheritance under polygenic control, yet their progeny means fell significantly below the mid-parent values, with no individuals surpassing the higher parent—a clear genetic tendency toward reduced fruit size (Table 2). The smaller fruit size of the paternal parent, combined with relatively limited additive gene interactions, likely accounts for this trend, consistent with observations in pear and Chinese cherry breeding [23].

Nutritional and flavor-related compounds presented a more complex genetic landscape. Total phenolic and anthocyanin contents exhibited high heritability, with substantial proportions of progeny exceeding the higher-parent value—evidence that favorable alleles for these traits operate additively, offering clear potential for cumulative genetic gain through phenotypic selection. Flavonoid and soluble sugar contents, however, displayed a maternal inheritance bias. Soluble sugar, in particular, showed low heritability and no transgressive segregation, suggesting possible involvement of dominant gene action. Breeding strategies targeting these compounds might therefore prioritize high-value parents as maternal donors or incorporate backcrossing schemes [24].

The solid–acid ratio (SSC/TA) further illustrated the genetic complexity underlying fruit quality. Its population mean fell below the mid-parent value, with a distinct phenotypic skew toward lower-ratio individuals (Table 3). This pattern aligns with the well-documented genetic antagonism and close linkage between QTLs governing sugar and acid accumulation in fruit crops—a dynamic often ascribed to competition for shared carbon precursors and coordinated biosynthetic regulation [25,26].

Drought tolerance in hybrid progeny emerges as a complex trait orchestrated by multiple physiological mechanisms [27]. Central to this resilience is photosynthetic stability, which underpins sustained carbon assimilation under water-limited conditions [28]. Among photosynthetic pigments, chlorophyll a and total chlorophyll exhibited high heritability and typical additive inheritance—characteristics conducive to effective phenotypic selection. Chlorophyll b, by contrast, displayed heterosis and transgressive segregation, suggesting that dominant allelic effects may shape the regulatory genes governing chlorophyll interconversion [29].

Within the antioxidant system, catalase (CAT) activity—critical for H_2_O_2_ scavenging and ROS homeostasis—stood out for its high heritability and pronounced heterosis in the F_1_ generation, demonstrating that hybridization can effectively bolster this key enzymatic defense [30]. Peroxidase (POD) activity followed a different trajectory, adhering to a low-parent inheritance pattern with no progeny exceeding the higher parent. Despite its high heritability, the low genetic transmission ability (Ta) of POD points to predominantly non-additive genetic control, likely arising from epistatic interactions between parental alleles. Reduced glutathione (GSH) content presented yet another pattern: skewed toward the lower parent yet retaining high heritability and substantial Ta, with several transgressive individuals observed—evidence of a genetic architecture that integrates both additive and non-additive effects (Table 6).

Correlation analysis of fruit quality traits uncovered distinct patterns of association. Titratable acidity (TA) exhibited a strong negative correlation with SSC/TA, capturing the core metabolic trade-off between sugar accumulation and acid degradation during fruit maturation—a dynamic that ultimately governs flavor balance [31]. Flavonoid content correlated negatively with soluble sugar levels, hinting at a potential resource allocation conflict between primary and secondary metabolic pathways [32,33]. Fruit size and shape traits, meanwhile, formed a positively intercorrelated network, indicating that a coordinated genetic program directs overall fruit development (Figure 5) [34].

Among drought tolerance traits, photosynthetic pigment components displayed strong positive intercorrelations, reflecting a synergistic response to stress [35]. The correlations among antioxidant parameters, in turn, delineate an integrated ROS-scavenging system that helps preserve cellular integrity and leaf water status [36]. Adding another layer of complexity, the significant negative correlation between proline and chlorophyll a contents supports a metabolic trade-off wherein resources—such as nitrogen—may be redirected from photosynthesis to osmotic adjustment under drought conditions (Figure 7) [37].

Proline accumulation under drought stress presents a well-documented interpretational challenge: it can signal either adaptive osmotic adjustment or stress-induced damage [38]. Principal component analysis offered a data-driven resolution. In PC3, proline loaded positively with RWC and POD (Table 7), delineating a coordinated physiological pattern wherein genotypes with higher PC3 scores simultaneously maintain water status and antioxidant defense—a combination characteristic of adaptive function rather than stress injury [39]. The seven elite genotypes naturally exemplified this adaptive pattern, all maintaining RWC near or above the population mean (54.91%) while keeping proline levels (103–282 μg·g^−1^) below the population mean (322 μg·g^−1^). H25, for instance, combined moderate proline (254.13 μg·g^−1^) with high RWC (60.61%) and elevated POD activity (147.22 U·g^−1^ FW). Such variation confirms that selection favored coordinated trait combinations over a simplistic proline threshold—a finding consistent with the current understanding that proline homeostasis and its interplay with cellular redox balance are more critical for stress tolerance than proline accumulation alone [40,41].

Principal component analysis (PCA) was employed in this study to construct integrated evaluation systems for fruit quality and drought tolerance, weighting traits objectively based on their inherent variance structure [42]. This framework enabled the identification of seven elite individuals—H3, H4, H8, H10, H11, H14, and H25—through a dual-trait selection strategy that simultaneously demanded high drought tolerance and superior fruit quality. These findings underscore the potential of interspecific recombination to overcome trait trade-offs and pyramid favorable alleles (Figure 8) [43], while simultaneously validating PCA as a powerful tool for screening superior genotypes.

Several methodological considerations warrant acknowledgment. The heritability estimates presented here derive from a single environment, with environmental variance calculated from parental replicates following standard quantitative genetics procedures. Should the genetically diverse F_1_ progeny prove more environmentally sensitive than their parents, this approach may underestimate true environmental variance, consequently overstating heritability. Hence, the heritability values reported should be interpreted as upper-limit estimates specific to the conditions of this trial [44].

Looking beyond the current study, the absence of multi-year validation leaves the stability of these elite genotypes across varying growing seasons an open question. Their fertility and cross-compatibility, likewise, demand further investigation before they can be fully deployed in subsequent breeding programs.

## 4. Materials and Methods

### 4.1. Plant Materials

An interspecific hybrid population was generated in 2021 by crossing the cultivated blackberry ‘Prime-Ark^®^ Freedom’ (female parent) with the wild species *Rubus chingii* (male parent). The resulting seeds were sown the following year, producing an F_1_ population of 108 seedlings. In 2023, these seedlings were transplanted to field conditions at the experimental station of the Institute of Botany, Jiangsu Province and Chinese Academy of Sciences (Nanjing, China), where they were cultivated for subsequent evaluation.

### 4.2. Analysis of Floral Morphological Traits of Rubus *spp.* × Rubus chingii Hybrids

Floral morphological traits were assessed during peak bloom (April–May 2025) across all 108 flowering individuals. Traits recorded included petal color, petal shape, petal diameter, and number of flowers per inflorescence. petal diameter was measured on fully opened flowers using a digital caliper (MNT-150, DEGUQMNT, Hanover, Germany; precision 0.01 mm). For each plant, observations were conducted on ten flowering branches, with three biological replicates per measurement.

### 4.3. Analysis of Leaf Morphological Traits of Rubus *spp.* × Rubus chingii Hybrids

Leaf trait measurements were performed during the vigorous growth stage. From each plant, the terminal leaflet of leaves positioned at the 3rd to 5th node on the primary stem was sampled. Leaf length (from base to tip) and maximum leaf width were recorded using a ruler, and the leaf shape index calculated as the length-to-width ratio. Leaf chlorophyll content was estimated with a SPAD-502 chlorophyll meter (Konica Minolta, Tokyo, Japan). All measurements were based on ten leaves per plant, with three biological replicates per trait.

### 4.4. Analysis of Fruit Appearance and Nutritional Quality Traits of Rubus *spp.* × Rubus chingii Hybrids

Fruit traits were evaluated at commercial maturity across 90 individuals, each selected for bearing at least five fruit-bearing branches. The experimental design followed a completely randomized arrangement within the field plot. For each genotype, all available uniformly developed, fully ripe fruits were collected from a minimum of five branches per plant. These collected fruits were subsequently divided randomly into three biological replicates, with each replicate containing approximately equal numbers of fruits—at least five per replicate whenever fruit availability permitted.

For morphological assessment, fruit diameter (FD) and fruit thickness (FT) were measured in millimeters using a digital caliper; the fruit shape index (FSI) was subsequently calculated as FD/FT. Single-fruit weight (SFW) was determined in grams using an electronic analytical balance (Jiangke Co., Shanghai, China; sensitivity 0.01 g), while fruit firmness (FF) was recorded in N with a GY-4 digital fruit firmness tester (TOP Instrument Co., Hangzhou, China). All morphological measurements were conducted on every fruit within each biological replicate, and replicate means were calculated for subsequent analyses.

Following morphological evaluation, fruits from each biological replicate were separately pooled, homogenized, and processed for biochemical assays. Soluble solids content (%) was measured from each replicate using a PAL-1 handheld refractometer (Atago, Guangzhou, China), with three technical replicates performed per biological replicate. For all remaining biochemical determinations, subsamples of homogenized tissue were taken—typically 10–50 g per replicate, depending on available fruit mass—and analyzed in triplicate.

Titratable acidity (%) was determined by acid–base potentiometric titration using a ZD-2 automatic titrator (Jinmai Instrument Co., Hangzhou, China), with 0.1 M NaOH titrated to pH 8.1 [45]. Total phenolic content (mg·g^−1^) was quantified via the Folin–Ciocalteu method, employing gallic acid as a standard and measuring absorbance at 765 nm [46]. Anthocyanin content (mg·g^−1^) was assessed using the pH differential method with buffers at pH 1.0 and 4.5, measuring absorbance at both 520 nm and 700 nm [47]. Vitamin C content (μg·g^−1^) was determined through Fe^3+^ reduction colorimetry at 536 nm, with ascorbic acid serving as the standard [48]. Soluble sugar content (μg·g^−1^) was measured using the anthrone-sulfuric acid method, with glucose as the standard and absorbance recorded at 620 nm [49]. Flavonoid content (mg·g^−1^) was evaluated by the aluminum nitrate colorimetric method, using rutin as the standard and measuring absorbance at 502 nm [50].

### 4.5. Evaluation of Drought Tolerance Traits of Rubus *spp.* × Rubus chingii Hybrids

A controlled drought stress treatment was imposed in the field during the vigorous shoot growth phase following fruit harvest in July 2025. Plants were arranged in a completely randomized design throughout the plot. Prior to treatment initiation, all 108 F_1_ individuals and both parental lines received initial irrigation to standardize soil moisture [51].

To establish uniform starting conditions—a fundamental requirement for reliable field drought screening [8]—all plants were irrigated to field capacity prior to stress initiation. This was achieved through a two-stage irrigation protocol. First, an evening application (after 18:00) continued until the root zone (approximately 30 cm × 30 cm) became visibly saturated, with standing water persisting for 30 min. A second irrigation followed the next morning (6:00–7:00) to compensate for overnight percolation, ensuring that all plants entered the stress period with fully recharged soil moisture.

To minimize the impact of inherent variability among F_1_ progeny, two complementary strategies were implemented. Standardized sampling was employed to reduce diurnal and positional variation: leaves were collected from the 4th to 5th fully expanded leaf from the shoot apex between 8:00 and 10:00 a.m., a time window selected specifically to capture stable water status measurements [52]. Furthermore, parental lines were interspersed throughout the plot, serving as internal benchmarks for physiological comparisons under identical growing conditions.

Following the initial irrigation protocol, water was withheld for seven consecutive days under natural rain-free conditions, with daytime temperatures frequently reaching 38 °C. This treatment duration was selected based on preliminary observations indicating that blackberry plants typically exhibit visible stress symptoms after 5–7 days without irrigation—a window that allows sufficient stress induction while avoiding plant mortality [9]. At the conclusion of the stress period, leaf samples were collected from the 3rd to 4th mature leaf from the shoot apex of each plant. For every genotype, three biological replicates were obtained, each sampled from a distinct branch. Samples were immediately placed in pre-chilled bags, maintained on ice, and transported to the laboratory for subsequent analysis [7].

A suite of physiological parameters was measured following established protocols, with three biological replicates per plant. Peroxidase (POD) activity was determined via the guaiacol method, monitoring the oxidation of guaiacol at 470 nm; results were expressed as U·g^−1^ FW [53]. Catalase (CAT) activity was assayed using the ammonium molybdate method, measuring H_2_O_2_ decomposition at 405 nm and reported as nmol·mg^−1^ prot [54]. Malondialdehyde (MDA) content was quantified through the thiobarbituric acid (TBA) reaction, with absorbance recorded at 532 nm (nmol·mg^−1^ prot) [55]. Proline (Pro) content was estimated by the acidic ninhydrin method, measuring absorbance at 520 nm and expressing results as μg·g^−1^ FW [56]. Reduced glutathione (GSH) content was measured via the DTNB method, monitoring the reaction with 5,5′-dithiobis- (2-nitrobenzoic acid) at 420 nm; values were reported as mg GSH·g^−1^ prot [57]. Chlorophyll a, chlorophyll b, and total chlorophyll contents were determined spectrophotometrically by measuring absorbance at 663 and 645 nm, with results expressed as mg·g^−1^ FW [58]. Relative water content (RWC) (%) was determined gravimetrically. Briefly, leaf discs were weighed for fresh weight (*Wf*), hydrated to full turgor for 12 h to obtain saturated weight (*Wt*), and oven-dried at 85 °C to constant dry weight (*Wd*). RWC was calculated as: *WC* = (*Wf* − *Wd*)/(*Wt* − *Wd*) × 100% [59]. 

### 4.6. Statistical Analysis

The data were managed in Microsoft Excel 2016, and the figures were generated via Origin 2022. Genetic parameters for major economic traits were calculated on the basis of established quantitative genetics methods. The following parameters were derived from parental and hybrid progeny data:

The Mean, Median parental value (MP), ratio of higher high parents (HH) between the two parents (BP), ratio of lower than low parents (LL), genetic transmitting ability (Ta), coefficient of variation (CV), environmental variance (Ve), and broad-sense heritability (H2) were calculated via a formula based on previously reported methods [60,61]. The relevant calculation formulas are as follows: (where P1 and P2 represent parental values; S denotes the standard deviation of hybrid progeny; F represents the mean value of hybrid progeny;$\;\sigma_{E}^{2}$ denotes the variance of parents; and $\sigma_{F}^{2}$ denotes the variance of hybrid progeny).

Parental mean (MP): MP=(P1+P2)/2.

Percentage of progeny exceeding the higher parent value (HH): HH = Number of super-high-parent plants/Total number of hybrid progeny × 100%.

Percentage of progeny with values between the two parents (BP): BP = (Total number of hybrid progeny—Number of super-high-parent plants—Number of below-low-parent plants)/Total number of hybrid progeny × 100%;

Percentage of progeny below the lower parent value (LL): LL = Number of plants below low-parity/Total number of hybrid progeny × 100%.

Coefficient of variation: $CV=\frac{S}{F}\times 100\%$.

Genetic transmission ability: $Ta=\frac{F}{MP}\times 100\%$.

Genetic transmission ability (Ta) reflects the capacity of parents to transmit their phenotypic traits to offspring [62,63], and has been widely used in fruit tree breeding to evaluate parental breeding value [60]. Its underlying theoretical basis—parent–offspring regression—is well established in classical quantitative genetics [64].

Environmental variance: $\sigma_{E}^{2}=\frac{\sigma_{P_{1}}^{2}+\sigma_{p_{2}}^{2}}{2}$.

Broad-sense heritability: $H^{2}=\frac{\sigma_{F}^{2}-\sigma_{E}^{2}}{\sigma_{F}^{2}}$.

Correlation analysis: To explore the intrinsic relationships between fruit quality and drought tolerance traits, Pearson correlation analysis was conducted on the 13 fruit traits and 9 drought tolerance traits. This analysis was performed in OriginPro 2024 to calculate the correlation coefficient (r) and corresponding *p*-value for each trait pair. Significance thresholds were defined as follows: *p* < 0.05 (significant, *), *p* < 0.01 (highly significant, **), and *p* < 0.001 (extremely significant, ***). A heatmap was generated from the correlation coefficient matrix to visually represent the results, with significance levels indicated by asterisks.

Principal component analysis: To account for differences in scale and units among traits, the raw data were standardized via the Z-score method, and each observation was transformed to a mean of 0 and a standard deviation of 1. The calculation was as follows:$z_{ij}=\frac{x_{ij}-{\bar{x}}_{j}}{S_{j}}$, where $z_{ij}$ is the standardized value for sample i and trait j; $x_{ij}$ is the original value; and ${\bar{x}}_{j}$ and $s_{j}$ are the mean and standard deviation of all samples for trait j, respectively. The standardized matrix Z was used as input for PCA in OriginPro 2024. Principal components were extracted on the basis of eigenvalues (λ > 1). Sample scores for each principal component were calculated as $F_{ik}=\sum_{j=1}^{P}u_{jk}z_{ij}$, where $u_{jk}$ is the loading of trait j on component k and *p* is the total number of traits. For fruit quality traits, the first four principal components (all eigenvalues > 1) were retained. For drought tolerance traits, the first three principal components (eigenvalues > 1) were retained, as the fourth component had an eigenvalue below 1.

The corresponding scores $F_{i1}$ to $F_{in}$ were derived. A linear weighted model was constructed for comprehensive evaluation: the weight for each principal component was $w_{k}=\frac{\lambda_{k}}{\sum_{t=1}^{4}\lambda_{t}}$, and the comprehensive score for each sample was $S_{i}=\sum_{k=1}^{4}w_{k}F_{ik}$ [65,66].

## 5. Conclusions

Systematic evaluation of the F_1_ progeny derived from cultivated blackberry ‘Prime-Ark^®^ Freedom’ and wild *Rubus chingii* revealed continuous phenotypic variation across growth, fruit quality, and drought tolerance traits—clear evidence of quantitative polygenic inheritance and substantial genetic diversity within the population. Inheritance patterns varied markedly by trait. Anthocyanin and total phenolic contents exhibited strong additive effects coupled with high heritability, positioning them as promising targets for breeding nutritionally enhanced fruits. Catalase activity demonstrated pronounced heterosis, while proline content uniquely combined exceptionally high heritability with broad genetic variance; together, these traits emerge as reliable early physiological markers for drought tolerance.

An integrated quantitative framework, developed through principal component analysis (PCA), enabled simultaneous assessment of fruit quality and drought tolerance. Applying this dual-trait screening strategy identified seven elite genotypes—H3, H4, H8, H10, H11, H14, and H25—that uniquely combine robust drought tolerance with superior fruit quality. These selections constitute a core germplasm resource for breeding high-quality, drought-tolerant blackberry cultivars. Looking forward, multi-environment validation of these elite genotypes and in-depth genetic analysis—including QTL mapping and transcriptomic studies—will be essential to accelerate their development into commercially viable cultivars.

## Figures and Tables

**Table 1:** Genetic parameters of leaf and floral morphological traits in the F_1_ population.

Traits	P1	P2	MP	Mean	Ta/%	H^2^	HH/%	LL/%	BP/%	CV/%
SPAD Value	32.25	36.00	34.13	30.72	90.01	0.512	6.48	72.22	21.30	9.53
Leaf length (cm)	7.50	7.04	7.27	5.88	80.88	0.993	7.41	23.15	69.44	17.36
Leaf width (cm)	5.08	6.12	5.60	4.29	76.61	0.984	23.15	6.48	70.37	20.16
Leaf shape index	1.50	1.17	1.34	1.41	105.22	0.991	16.67	71.29	12.04	23.59
Petal diameter (mm)	45.0	41.0	43.0	40.8	94.88	0.987	25.93	28.70	45.37	15.23
Number of flowers per inflorescence	3.25	1.25	2.25	1.61	71.56	0.488	0.93	20.37	78.7	37.68

Note: P1 and P2: phenotypic values of the cultivated parent ‘Prime-Ark^®^ Freedom’ and the wild parent *Rubus chingii*, respectively; MP: mid-parent value; Mean: mean of the F_1_ population; Ta: genetic transmission ability; H^2^: broad-sense heritability; HH, LL, BP: percentage of progeny with phenotypic values exceeding the higher parent, below the lower parent, and between the two parents, respectively; CV: coefficient of variation.

**Table 2:** Genetic parameters of fruit appearance traits in the F_1_ population.

Traits	P1	P2	MP	Mean	Ta/%	H^2^	HH/%	LL/%	BP/%	CV/%
Single fruit weight (g)	13.33	2.27	7.80	4.21	53.97	0.79	0.00	28.57	71.43	56.60
Fruit diameter (mm)	25.49	15.67	20.58	17.94	87.17	0.69	1.06	21.28	77.66	14.61
Fruit thickness (mm)	36.99	18.43	27.71	20.90	75.42	0.83	0.00	41.67	58.33	24.01
Fruit shape index	1.45	1.18	1.32	1.37	103.79	0.54	8.51	32.22	59.27	16.05
Fruit firmness (N)	0.83	2.44	1.64	1.04	63.41	0.33	0.00	9.89	90.11	16.99

Note: P1 and P2: phenotypic values of the cultivated parent ‘Prime-Ark^®^ Freedom’ and the wild parent *Rubus chingii*, respectively; MP: mid-parent value; Mean: mean of the F_1_ population; Ta: genetic transmission ability; H^2^: broad-sense heritability; HH, LL, BP: percentage of progeny with phenotypic values exceeding the higher parent, below the lower parent, and between the two parents, respectively; CV: coefficient of variation.

**Table 3:** Genetic parameters of fruit quality traits in the F_1_ population.

Traits	P1	P2	MP	Mean	Ta/%	H^2^	HH/%	LL/%	BP/%	CV/%
Soluble sugar content (μg·g^−1^)	971.89	4194.40	2583.15	1499.94	58.07	0.995	0.00	14.44	85.56	27.17
Soluble solid content (%)	11.52	11.32	11.42	11.19	98.06	0.997	34.44	54.44	11.12	9.05
TA content (%)	0.98	1.31	1.15	1.41	122.61	0.999	57.78	12.22	30.00	26.15
SSC/TA	11.82	8.68	10.25	8.51	83.09	0.999	8.89	58.89	32.22	27.91
Vitamin C content (μg·g^−1^)	351.68	336.20	343.94	340.11	98.98	0.998	27.78	35.56	36.66	8.21
Total phenolic content (mg·g^−1^)	2.44	2.38	2.41	2.44	101.24	0.983	45.56	16.67	37.77	5.51
Anthocyanin content (mg·g^−1^)	1.74	0.86	1.30	1.81	139.23	0.892	47.78	0.00	52.22	19.72
Flavonoid content (mg·g^−1^)	0.83	1.25	1.04	0.93	89.42	0.998	10.00	40.00	50.00	33.56

Note: P1 and P2: phenotypic values of the cultivated parent ‘Prime-Ark^®^ Freedom’ and the wild *Rubus chingii*, respectively; MP: mid-parent value; Mean: mean of the F_1_ population; Ta: genetic transmission ability; H^2^: broad-sense heritability; HH, LL, BP: percentage of progeny with phenotypic values exceeding the higher parent, below the lower parent, and between the two parents, respectively; CV: coefficient of variation.

**Table 4:** Eigenvalues, variance contribution rates, and variable loadings from principal component analysis.

Traits	PC1	PC2	PC3	PC4
FL	0.0003	−0.3963	0.1398	−0.3139
AN	0.1642	0.1520	−0.2026	0.1566
TP	−0.0363	−0.1068	0.2194	0.7244
SS	−0.0031	0.1233	−0.2185	−0.1712
VC	−0.1520	0.0128	0.4452	0.0764
SSC/TA	−0.3068	0.5192	0.0246	0.1567
SFW	0.3118	0.0447	−0.1524	0.4888
FF	−0.0411	0.1907	0.6663	−0.0825
TA	0.3278	−0.4607	−0.0305	0.1190
SSC	−0.1671	0.3080	−0.3177	−0.0303
FSI	0.3894	0.1965	−0.0818	−0.0795
FD	0.4435	0.2822	0.1828	−0.0975
FT	0.5214	0.2447	0.1886	−0.1263
Variance Contribution Rate/%	19.9397	15.4897	10.4560	10.0772
Cumulative variance contribution rate/%	19.9397	35.4294	45.8854	55.9626
Eigenvalue (λ)	2.5922	2.0137	1.3593	1.3100

Note: FL, flavonoid content; AN, anthocyanin content; TP, total phenolic content; SS, soluble sugar content; VC, vitamin C content; TA, titratable acid content; SSC/TA, solid–acid ratio; SSC, soluble solids content; SFW, single-fruit weight; FF, fruit firmness; FSI, fruit shape index; FD, fruit diameter; FT, fruit thickness.

**Table 5:** Individual plants were ground on the basis of fruit quality evaluation.

Grade	Number of Progenies	Proportion (%)	Raw Score Range	Representative Plants
Superior	18	20	1.705~0.731	44, 74, 29, 17, 10, 59, 12, 20, 4, 47, 22, 9, 43, 3, 7, 86, 11, 60
Good	27	30	0.729~−0.053	78, 50, 38, 30, 62, 54, 81, 8, 31, 72, 35, 89, 68, 28, 52, 79, 48, 13, 18, 1, 61, 40, 15, 14, 39, 49, 58
Medium	27	30	−0.105~−0.643	53, 66, 21, 25, 45, 67, 27, 32, 6, 64, 76, 16, 46, 51, 65, 63, 41, 19, 26, 2, 83, 87, 37, 55, 5, 42, 73
Poor	18	20	−0.665~−1.923	85, 90, 23, 33, 34, 57, 24, 56, 80, 36, 75, 69, 70, 84, 77, 71, 88, 82

**Table 6:** Genetic parameters of physiological traits under drought stress in the F_1_ population.

Traits	P1	P2	MP	Mean	Ta/%	H^2^	HH/%	LL/%	BP/%	CV/%
Chlorophyll a (mg·g^−1^ FW)	1.44	3.49	2.47	1.69	68.46	0.75	0.00	23.15	76.85	20.52
Chlorophyll b (mg·g^−1^ FW)	1.39	2.46	1.93	1.98	102.77	0.96	18.52	16.67	64.81	29.20
Chlorophyll (mg·g^−1^ FW)	2.74	5.94	4.34	3.65	84.12	0.90	0.00	15.74	84.26	24.80
Relative water content (%)	59.80	72.05	65.93	54.91	83.30	0.86	6.48	68.52	25.00	18.49
Proline (μg·g^−1^ FW)	217.76	758.57	488.17	322.55	66.07	0.99	9.26	0.00	90.74	88.04
Peroxidase activity (U·g^−1^ FW)	122.44	434.84	278.64	97.34	34.94	0.91	0.00	73.15	26.85	45.30
Catalase activity (U·g^−1^ FW)	271.93	416.65	344.29	350.70	101.86	0.98	24.07	20.37	55.56	26.86
Content of reduced glutathion (mgGSH g^−1^prot)	3.11	3.76	3.44	2.90	84.36	0.92	14.81	62.04	23.15	27.01
Malondialdehyde (nmol·mg^−1^prot)	342.16	214.70	278.43	242.83	87.22	0.91	3.70	12.96	83.33	20.01

Note: P1 and P2: phenotypic values of the cultivated parent ‘Prime-Ark^®^ Freedom’ and the wild parent *Rubus chingii*, respectively; MP: mid-parent value; Mean: mean of the F_1_ population; Ta: genetic transmission ability; H^2^: broad-sense heritability; HH, LL, BP: percentage of progeny with phenotypic values exceeding the higher parent, below the lower parent, and between the two parents, respectively; CV: coefficient of variation. Proline accumulation can reflect either adaptive osmotic adjustment or stress damage. The genetic parameters presented describe phenotypic segregation patterns only and do not imply directional superiority.

**Table 7:** Eigenvalues, variance contribution rates, and variable loadings from principal component analysis of drought tolerance traits.

Traits	PC1	PC2	PC3
POD	0.1727	0.1929	0.5226
MDA	0.0461	−0.3766	0.0194
CAT	0.0166	0.5952	−0.3286
Pro	−0.1813	0.1974	0.4134
Chlorophyll	0.5708	0.0112	−0.0195
Chl a	0.5463	0.0086	−0.0696
Chl b	0.5556	0.0063	0.0172
GSH	−0.0316	0.5260	−0.3684
RWC	0.0310	0.3883	0.5535
Variance Contribution Rate/%	33.1460	14.7618	13.4371
Cumulative variance contribution rate/%	33.1460	47.9078	61.3449
Eigenvalue (λ)	2.9831	1.3286	1.2093

Note: POD, peroxidase activity; MDA, malondialdehyde content; CAT, catalase activity; Pro, proline content; Chlorophyll, total chlorophyll content; Chl a, chlorophyll a content; Chl b, chlorophyll b content; GSH, reduced glutathione content; RWC, relative water content.

**Table 8:** Main traits of selected superior plants in fruit quality and drought tolerance.

Plant-id	Total Phenolic Content (mg·g^−1^)	SSC/TA	Single Fruit Weight (g)	Fruit Firmness (N)	Relative Water Content (%)	Malondialdehyde Content (nmol·mg^−1^prot)	Catalase Activity (U·g^−1^ FW)	Chlorophyll Content (mg·g^−1^ FW)	Peroxidase Activity (U·g^−1^ FW)
H3	2.46	8.49	10.05	0.91	59.92	337.55	340.15	4.38	93.69
H4	2.51	7.47	6.61	1.27	65.53	225.31	393.07	3.63	120.72
H8	2.51	8.58	6.04	1.03	63.91	345.13	422.21	5.65	90.33
H10	2.44	7.72	5.53	1.11	61.08	208.14	338.57	3.05	60.5
H11	2.45	8.31	8.31	1.01	58.86	223.93	332.80	3.33	101.97
H14	2.54	7.54	6.54	1.31	52.36	276.64	345.92	3.78	110.01
H25	2.50	8.11	8.78	1.02	60.61	213.41	424.36	3.72	147.22

## Data Availability

The data supporting the results in this study are included within the article.

## References

[1] Strik B.C., Finn C.E., Clark J.R., Bañados M.P. (2008). Worldwide Production of Blackberries. Acta Hortic..

[2] Du L., Lü H., Chen Y., Yu X., Jian T., Zhao H., Wu W., Ding X., Chen J., Li W. (2023). Blueberry and blackberry anthocyanins ameliorate metabolic syndrome by modulating gut microbiota and short-chain fatty acids metabolism in high-fat diet-fed C57BL/6J Mice. J. Agric. Food Chem..

[3] Zannou O., Koca I. (2022). Greener extraction of anthocyanins and antioxidant activity from blackberry (*Rubus* spp.) using natural deep eutectic solvents. LWT-Food Sci. Technol..

[4] Siriwoharn T., Wrolstad R.E., Finn C.E., Pereira C.B. (2004). Influence of cultivar, maturity, and sampling on blackberry (*Rubus* L. Hybrids) anthocyanins, polyphenolics, and antioxidant properties. J. Agric. Food Chem..

[5] Du X.F., Kurnianta A., McDaniel M., Finn C.E., Qian M.C. (2010). Flavour profiling of ‘Marion’ and thornless blackberries by instrumental and sensory analysis. Food Chem..

[6] Ren B.R., Wang J.G., Gu R.S. (2001). Adaptability of blackberry to water, salt and low temperature stress. J. Plant Resour. Environ..

[7] Yang H.Y., Liu H., Wu W.L., Li W.L., Lyu L.F. (2020). Drought-Induced oxidative damage and antioxidant responses in blackberry cultivar ‘Hull Thornless’. Pak. J. Agric. Res..

[8] Yang H.Y., Zhang C.H., Wu W.L., Li W.L., Wei Y.L., Dong S.S. (2015). Physiological responses of blackberry cultivar ‘Ningzhi 1’ to drought stress. Russ. J. Plant Physiol..

[9] Zhang C., Yang H., Wu W., Li W. (2017). Effect of drought stress on physiological changes and leaf surface morphology in the blackberry. Braz. J. Bot..

[10] Wu W.L., Li W.L. (2005). Introduction, Cultivation and Utilization of Blackberry.

[11] Foster T.M., Bassil N.V., Dossett M., Worthington M.L., Graham J. (2019). Genetic and genomic resources for *Rubus* breeding: A roadmap for the future. Hortic. Res..

[12] Clark J.R., Finn C.E. (2008). New trends in blackberry breeding. Acta Hortic..

[13] Finn C.E., Clark J.R., Badenes M.L., Byrne D.H. (2012). Blackberry. Fruit Breeding.

[14] Sheng J.Y., Wang S.Q., Liu K.H., Zhu B., Zhang Q.Y., Qin L.P., Wu J.J. (2020). *Rubus chingii* Hu: An overview of botany, traditional uses, phytochemistry, and pharmacology. Chin. J. Nat. Med..

[15] Mikulic-Petkovsek M., Schmitzer V., Slatnar A., Stampar F., Veberic R. (2012). Composition of sugars, organic acids, and total phenolics in 25 wild or cultivated berry species. J. Food Sci..

[16] Yan C.X., Shao X.M., Ding X.Q., Xia Y., Sun C.Q., Liu C.H. (2015). Analysis of anatomical structure and characteristics of vegetative organs of *Rubus chingii* Hu. N. Hortic..

[17] Cortés A.J., López-Hernández F. (2021). Harnessing crop wild diversity for climate change adaptation. Genes.

[18] Dai A. (2013). Erratum: Increasing drought under global warming in observations and models. Nat. Clim. Change.

[19] Crosatti C., Quansah L., Maré C., Giusti L., Roncaglia E., Atienza S.G., Cattivelli L., Fait A. (2013). Cytoplasmic genome substitution in wheat affects the nuclear-cytoplasmic cross-talk leading to transcript and metabolite alterations. BMC Genom..

[20] Holland J.B., Nyquist W.E., Cervantes-Martínez C.T., Janick J. (2002). Estimating and interpreting heritability for plant breeding: An update. Plant Breeding Reviews.

[21] Piepho H.P., Möhring J., Melchinger A.E., Büchse A. (2008). BLUP for phenotypic selection in plant breeding and variety testing. Euphytica.

[22] Wang G., Gao X., Wang X., Liu P., Guan S.L., Qi K., Zhang S., Gu C. (2022). Transcriptome analysis reveals gene associated with fruit size during fruit development in pear. Sci. Hortic..

[23] Wang Y., Liu Z., Zhang J., Yang P., Ma L., Wang Z., Tu H., Yang S., Wang H., Chen T. (2022). Genetic tendency analysis of several floral and fruit traits in F1 hybrids of Chinese cherry. Hortic. Plant J..

[24] Wang N., Jiang S., Zhang Z., Fang H., Xu H., Wang Y., Chen X. (2018). *Malus sieversii*: The origin, flavonoid synthesis mechanism, and breeding of red-skinned and red-fleshed apples. Hortic. Res..

[25] Chen J., Wang N., Fang L.C., Liang Z.C., Li S.H., Wu B.H. (2015). Construction of a high-density genetic map and QTLs mapping for sugars and acids in grape berries. BMC Plant Biol..

[26] Gracia C., Calle A., Gasic K., Arias E., Wünsch A. (2025). Genetic and QTL analyses of sugar and acid content in sweet cherry (*Prunus avium* L.). Hortic. Res..

[27] Chaves M.M., Maroco J.P., Pereira J.S. (2003). Understanding plant responses to drought—From genes to the whole plant. Funct. Plant Biol..

[28] Chaves M.M., Flexas J., Pinheiro C. (2009). Photosynthesis under drought and salt stress: Regulation mechanisms from whole plant to cell. Ann. Bot..

[29] Tanaka R., Tanaka A. (2007). Tetrapyrrole biosynthesis in higher plants. Annu. Rev. Plant Biol..

[30] Miller G., Suzuki N., Ciftci-Yilmaz S., Mittler R. (2010). Reactive oxygen species homeostasis and signalling during drought and salinity stresses. Plant Cell Environ..

[31] Sweetman C., Deluc L.G., Cramer G.R., Ford C.M., Soole K.L. (2009). Regulation of malate metabolism in grape berry and other developing fruits. Phytochemistry.

[32] Liu S., An X., Xu C., He D., Li X., Chen C., Guo B., Xu D., Huang J. (2025). Integrative transcriptomic-physiological analysis deciphers nitrogen-mediated carbon reallocation balancing growth and flavonoid metabolism in *Epimedium pubescens*. Front. Plant Sci..

[33] Zhang X., Zhang D., Li W., Li J., Li S., Zhang W., Zhang P., Cui K., Huo J., Gang H. (2025). Transcriptome profiling reveals the regulatory mechanisms of AsA (ascorbic acid) and flavonoid synthesis and metabolic processes in fruit development of *Ribes nigrum* L. Mol. Genet. Genom..

[34] Tanksley S.D. (2004). The genetic, developmental, and molecular bases of fruit size and shape variation in tomato. Plant Cell.

[35] Tanaka A., Tanaka R. (2006). Chlorophyll Metabolism. Curr. Opin. Plant Biol..

[36] Nadarajah K.K. (2020). ROS homeostasis in abiotic stress tolerance in plants. Int. J. Mol. Sci..

[37] Kavi Kishor P.B., Sreenivasulu N. (2014). Is proline accumulation perse correlated with stress tolerance or is proline homeostasis a more critical issue?. Plant Cell Environ..

[38] El Moukhtari A., Cabassa-Hourton C., Farissi M., Savouré A. (2022). Contribution of Exogenous Proline to Abiotic Stresses Tolerance in Plants: A Review. Int. J. Mol. Sci..

[39] Yan Z.M., Sun J., Guo S.R., Wei H., Su Y.C., Wang C. (2014). Effects of Exogenous Proline on Ascorbate-Glutathione Cycle in Melon Seedling Roots under Salt Stress. Zhiwu Kexue Xuebao.

[40] Kaur G., Asthir B. (2015). Proline: A Key Player in Plant Abiotic Stress Tolerance. Biol. Plant..

[41] Renzetti M., Funck D., Trovato M. (2024). Proline and ROS: A Unified Mechanism in Plant Development and Stress Response?. Plants.

[42] Jolliffe I.T., Cadima J. (2016). Principal Component Analysis: A review and recent developments. Philos. Trans. R. Soc. A—Math. Phys. Eng. Sci..

[43] Nowicka P., Wojdyło A., Laskowski P. (2019). Principal component analysis (PCA) of physicochemical compounds’ content in different cultivars of peach fruits, including qualification and quantification of sugars and organic acids by HPLC. Eur. Food Res. Technol..

[44] Roka P., Shrestha S., Adhikari S.P., Neupane A., Shreepaili B., Bista M.K. (2024). A review on genetic parameters estimation, trait association, and multivariate analysis for crop improvement. Arch. Agric. Environ. Sci..

[45] Etienne A., Génard M., Bancel D., Benoit S., Nonone M., Barre F., Bugaud C. (2015). Modeling changes in pH and titratable acidity during the maturation of dessert banana. Acta Hortic..

[46] Zawawi N., Chong P.J., Mohd Tom N.N., Saiful Anuar N.S., Mohammad S.M., Ismail N., Jusoh A.Z. (2021). Establishing relationship between vitamins, total phenolic and total flavonoid content and antioxidant activities in various honey Types. Molecules.

[47] Cheng F.R., Cui H.X., Fang J.L., Yuan K., Jin S.H., Zhu X.T., Xu Y. (2021). Content determination of functional composition and antioxidant activity from six purple plants. Pharmacogn. Mag..

[48] Kampfenkel K., Vanmontagu M., Inze D. (1995). Extraction and determination of ascorbate and dehydroascorbate from plant tissue. Anal. Biochem..

[49] DuBois M., Gilles K.A., Hamilton J.K., Rebers P.A., Smith F. (1956). Colorimetric method for determination of sugars and related substances. Anal. Chem..

[50] Zhishen J., Mengcheng T., Jianming W. (1999). The determination of flavonoid contents in mulberry and their scavenging effects on superoxide radicals. Food Chem..

[51] Osmolovskaya N., Shumilina J., Kim A., Didio A., Grishina T., Bilova T., Tarakhovskaya E., Zhukov V., Tikhonovich I., Frolov A. (2018). Methodology of Drought Stress Research: Experimental Setup and Physiological Characterization. Int. J. Mol. Sci..

[52] Wigley B.J., Charles-Dominique T., Hempson G.P., Stevens N., TeBeest M., Archibald S., Bond W.J., Bunney K., Coetsee C., Donaldson J. (2020). A Handbook for the Standardised Sampling of Plant Functional Traits in Disturbance-Prone Ecosystems, with a Focus on Open Ecosystems. Aust. J. Bot..

[53] Fielding J.L., Hall J.L. (1978). A biochemical and cytochemical study of peroxidase activity in roots of Pisum sativum: I. A comparison of DAB-peroxidase and guaiacol-peroxidase with particular emphasis on the properties of cell wall activity. J. Exp. Bot..

[54] Aebi H. (1984). Catalase in vitro. Methods in Enzymology.

[55] Bailly C., Benamar A., Corbineau F., Come D. (1996). Changes in malondialdehyde content and in superoxide dismutase, catalase and glutathione reductase activities in sunflower seeds as related to deterioration during accelerated aging. Physiol. Plant..

[56] Bates L.S., Waldren R.P., Teare I.D. (1973). Rapid determination of free proline for water-stress studies. Plant Soil.

[57] Griffith O.W. (1980). Determination of glutathione and glutathione disulfide using glutathione reductase and 2-vinylpyridine. Anal. Biochem..

[58] Lichtenthaler H.K., Wellburn A.R. (1983). Determinations of total carotenoids and chlorophylls a and b of leaf extracts in different solvents. Biochem. Soc. Trans..

[59] Turner N.C. (1981). Techniques and experimental approaches for the measurement of plant water status. Plant Soil.

[60] Falconer D.S., Mackay T.F.C. (1996). Introduction to Quantitative Genetics.

[61] Lippman Z., Tanksley S.D. (2001). Dissecting the genetic pathway to extreme fruit size in tomato using a cross between the small-fruited wild species *Lycopersicon pimpinellifolium* and *L. esculentum* var. Giant Heirloom. Genetics.

[62] Pei H.S. (1964). A Preliminary Account on a Theory of Relative Heritability (A Mathematical Analysis for Dominant and Segregation of Hybrids According to the Relative Intensity of Heredity). Acta Agron. Sin..

[63] Liu L.F., Mao S.X., Huang Y.Z. (1984). Quantitative Genetics in Crops.

[64] Liu J.C., Xu M., Han Y., Liu W.S. (2024). Genetic Analysis of Fruit Economic Traits in F_1_ Population of ‘Chuanzhihong’ × ‘Katy’ Apricot. N. Fruits.

[65] Greenacre M., Groenen P.J.F., Hastie T., D’Enza A.I., Markos A., Tuzhilina E. (2022). Principal component analysis. Nat. Rev. Methods Primers.

[66] Hotelling H. (1933). Analysis of a complex of statistical variables into principal components. J. Educ. Psychol..

